# Beyond a reference genome: pangenomes and population genomics of underutilized and orphan crops for future food and nutrition security

**DOI:** 10.1111/nph.18021

**Published:** 2022-03-22

**Authors:** Mark A. Chapman, Yuqi He, Meiliang Zhou

**Affiliations:** ^1^ Biological Sciences University of Southampton Life Sciences Building 85, Highfield Campus Southampton SO17 1BJ UK; ^2^ Institute of Crop Sciences Chinese Academy of Agricultural Sciences Room 405, National Crop Gene Bank Building Zhongguancun South Street No. 12 Haidian District Beijing 100081 China

**Keywords:** crop improvement, food security, genomics, pangenome, population genomics, reference genome, underutilized crops

## Abstract

Underutilized crops are, by definition, under‐researched compared to staple crops yet come with traits that may be especially important given climate change and the need to feed a globally increasing population. These crops are often stress‐tolerant, and this combined with unique and beneficial nutritional profiles. Whilst progress is being made by generating reference genome sequences, in this Tansley Review, we show how this is only the very first step. We advocate that going ‘beyond a reference genome’ should be a priority, as it is only at this stage one can identify the specific genes and the adaptive alleles that underpin the valuable traits. We sum up how population genomic and pangenomic approaches have led to the identification of stress‐ and disease‐tolerant alleles in staple crops and compare this to the small number of examples from underutilized crops. We also demonstrate how previously underutilized crops have benefitted from genomic advances and that many breeding targets in underutilized crops are often well studied in staple crops. This cross‐crop population‐level resequencing could lead to an understanding of the genetic basis of adaptive traits in underutilized crops. This level of investment may be crucial for fully understanding the value of these crops before they are lost.

**Table:** 

	**Contents**	
	Summary	1583
I.	Introduction	1583
II.	Genomics of underutilized crops to‐date	1585
III.	The way forward	1589
	Acknowledgements	1592
	References	1592

## I. Introduction

In 2020, at least 720 million people (≥ 9.9% of the world’s population) faced hunger, an increase on previous years, and the greatest percentage of the total population since 2010 (FAO *et al*., 2021). With ongoing climate change, the increasing global population and the COVID19 pandemic, the number of people facing hunger is expected to rise significantly. It is increasingly recognized that achieving Sustainable Development Goal (SDG) 2 (‘Zero Hunger’) by 2030 is in doubt (Global Panel on Agriculture & Food Systems for Nutrition, 2020). To overcome this challenge, one of the most favourable approaches is to breed new germplasm to face these stresses. Crop breeders have identified germplasm (either local varieties or crop wild relatives (CWRs)) with beneficial traits, and introgressed the adaptive alleles into elite varieties. Although substantial efforts and many successes have been made to breed climate‐resilient crops, yield has started to plateau because of negative effects of extreme weather events in at least some years and in some parts of the world (Zhao *et al*., 2017). Homogenization of diets and the increasing consumption of calorie‐rich yet nutrition‐deficient crops has also led to an increase in hidden hunger, a significant factor threatening human health (Khoury *et al*., 2014; Dawson *et al*., 2019). A greater understanding of the effect of environmental stresses on crop quality, for example nutrient content and composition, is also needed.

Staple crops are limited in their tolerance of a changing climate, forcing researchers and breeders to start to investigate new pathways to ensure future food security. Underutilized crops (sometimes known as semidomesticated, orphan and/or forgotten crops) are locally important crops grown in limited regions with low‐input conditions. They are currently impossible or inefficient to be produced at large scale due to low yield, antinutrient content, consumer preferences, harvestability or other properties. Because of the diverse nutrient and local adaptations, these crops are often locally important or vital, and represent a broad gene pool for future crop improvement. Examples include high iron, zinc and calcium content in tepary bean and bambara groundnut (Chibarabada *et al*., 2017), tolerance of drought stress in lablab, horsegram and cowpea (Muchow, 1985; Ewansiha & Singh, 2006), and tolerance of heat in amaranth and cassava (Kuo *et al*., 1992). Thus, underutilized crops have significant potential to aid food security (Massawe *et al*., 2016; Mabhaudhi *et al*., 2017; X. Li *et al*., 2020; Siddique *et al*., 2021). However, some underutilized crops are becoming abandoned due to globalization, migration and other economic forces and therefore are at risk of loss of diversity or complete loss of the crop, or the loss of associated indigenous knowledge (Mabhaudhi *et al*., 2017).

This review will summarize advances made in major crops, and how genome sequencing has brought about a step‐change in agricultural research. We also discuss some crops which could have been described as underutilized only *c*. 30 yr ago but have become more mainstream, and the genomics‐assisted research that has taken place. Beyond this, we discuss the benefits of venturing ‘beyond a reference genome’ (i.e. resequencing, population genomics and pangenomics) and what advantages this can bring. We encourage the research community to incorporate underutilized crops into wider research and to envisage the benefits that come from this increased investment.

### 1. Genomics and its contribution to agricultural improvements

For staple crops, notable successes have been made using genomic‐related approaches to uncover the genes responsible for important phenotypes.

#### Genome‐wide association study

Genome‐wide association study (GWAS) relies on genome‐scale data (dense single nucleotide polymorphisms (SNPs) or whole genome sequences) to associate genomic variation with trait variation (Liu & Yan, 2019). Typically, the analysis of hundreds of varieties/lines is necessary to generate sufficient power to resolve quantitative trait loci (QTL), as well as to ensure rare causative variants are included (e.g. Xing *et al*., 2015). This breadth of variation encompasses orders of magnitude more recombination than QTL mapping and therefore can resolve even small‐effect QTL and with typically greater resolution. Thousands of GWAS have been reported and do not just cover the staple cereals, such as cucumber, peach, sesame and lettuce (Liu & Yan, 2019), but GWAS of crops that fit the definition of underutilized are in their infancy (see the Section ‘Population genomics in underutilized crops’).

#### Nested‐association mapping

Nested‐association mapping (NAM) involves the development of multiple mapping populations sharing one parent, circumventing in part the issues related to traditional QTL mapping approaches: only covering the variation present in two parents and the low power to tightly define QTL due to limited recombination events (McMullen *et al*., 2009). With this increased power (Bouchet *et al*., 2017), resources are being developed for a range of crops, and have provided candidate genes for a range of agronomic traits, for example flowering time in maize (Buckler *et al*., 2009), barley (Maurer *et al*., 2015) and sorghum (Bouchet *et al*., 2017), and plant architectural traits in maize (Brown *et al*., 2011) and durum wheat (Kidane *et al*., 2019). Many of these studies have confirmed previous work as well as identified novel QTL for follow up, but are currently restricted to only the main crops.

#### Pangenomes

It has become increasingly clear that across varieties of a crop, presence/absence variation is common; this means that any one reference genome contains only a subset of the species’ genome, potentially lacking causative loci (Della Coletta *et al*., 2021). Sequencing and assembling multiple varieties and adding novel genomic regions to the reference results in the pangenome. Genes present in all accessions are defined as ‘core’ and others as ‘dispensable’. In some cases, half or less of some crop genomes are core (Gordon *et al*., 2017; Wang *et al*., 2018; Haberer *et al*., 2020), although these values are highly dependent on the number of accessions sequenced, and whether wild relatives are examined. This rare variation can underlie loci of agronomic importance, for example a flavour‐related gene in tomato (Gao *et al*., 2019). Pangenomics has also revealed some important evolutionary genomic insight; core maize genes exhibit much greater expression than dispensable genes (Haberer *et al*., 2020), and dispensable regions tend to have higher transposable element (TE) content than core regions (e.g. Gao *et al*., 2019).

Most of the above studies have focused on agronomic traits such as yield and its associated components (flowering time, plant stature and size), but because of ensuing climate change, a concerted effort to increase the study of climate change resilience phenotypes, for example root traits and stress response, would be prudent. Whilst some studies have investigated this, for example using NAM to explore drought‐adaptive phenotypes in barley (Pham *et al*., 2019) and maize (Li *et al*., 2016), these are comparatively rare.

### 2. Genome‐assisted advances in staple crops

Here we focus on rice and maize as two of the most widely grown staple crops, collectively grown on 360 million ha in 2019 (http://faostat.fao.org; accessed August 2021), to highlight how genomic advances have led to significant crop improvement for hundreds of millions of consumers.

#### Genomic advances in rice

Rice is one of the most important crops providing staple food for more than half of the world’s population. Due to the relatively small genome size (430 Mb), colinearity with other cereals, a highly efficienct genetic transformation system and abundant genetic resources, rice has become a model grass species equivalent to the eudicot *Arabidopsis*. Significant efforts have been made to assemble and annotate the rice genome, for the *japonica* subspecies (cv Nipponbare; Goff *et al*., 2002) and the *indica* subspecies (93‐11; Yu *et al*., 2002). Compared to *Arabidopsis*, rice exhibits a gradient in GC content, which means a large proportion of rice genes have no obvious homologue in *Arabidopsis* (Yu *et al*., 2002).

Rice is an excellent candidate for population genomic analysis because of its extremely strong population structure and the large extent of linkage disequilibrium (LD) owing to self‐pollination. Resequencing of 527 rice landraces clearly separated the two cultivated subspecies and further divided these by latitudinal photoperiod and temperature clines (Huang *et al*., 2010). Genome analysis of 446 wild rice (*Oryza rufipogon*) accessions and 1083 cultivated varieties identified a population of *O*. *rufipogon* in southern China where it appears that the domestication into the *japonica* subspecies took place; from there, hybridization with local wild rice formed *indica* rice, which spread into Southeast and South Asia (Huang *et al*., 2012). By combining genomic and phenotypic data through GWAS, a variety of loci associated with agronomic traits in rice have been identified (Supporting Information Table S1).

Whilst mapping short sequence reads of other varieties onto one of the reference genomes can allow these types of analyses, this means that genomic regions absent from the reference will be ignored. Therefore, more recently the genomes of other varieties with beneficial agronomic traits (Zhao *et al*., 2018; Choi *et al*., 2020), weedy rice (Sun *et al*., 2019) and other species of *Oryza* (W. Li *et al*., 2020) have been sequenced and assembled, forming a potential rice pangenome resource. A rice pangenome initiative, involving the comparison of 16 *de novo* assembled genomes from the main population genomic groups (sequenced and assembled using long reads and optical mapping, and hence are described as ‘platinum’ genomes), showed that an average of 33.7 Mb of genome was absent among all pairwise comparisons (Zhou *et al*., 2020). This highlights how using one reference will only ever describe variation in a subset of the entire crop’s (pan)genome. It is worth noting that the majority (nearly 90%) of the presence/absence fraction of the rice pangenome is made up of TEs, indicating these are more evolutionary labile, but the remaining 10% contains potentially protein‐coding loci. These genome resources will promote evolutionary studies and the identification of adaptive genetic variation in rice.

#### Genomic advances in maize

Maize was domesticated from teosinte in southwestern Mexico *c*. 10 000 yr ago. From its wild progenitor, maize has evolved a strikingly different morphology, forming an unbranched plant with large cobs and sweet, naked kernels (Doebley, 1990; Matsuoka *et al*., 2002). Since then, maize has been continuously improved, and an array of hybrid lines suitable for modern agricultural practice have been developed. In the past century, maize yield has increased eight‐fold due to the increase in yield per plant and plant density adapted by harnessing heterosis. The ancestor genome of maize experienced a tetraploid intermediate stage (*n* = 20), and then a series of chromosome fusions led to diploidization and recovery of the chromosome number *n* = 10 (Schnable, 2015). Thus, the maize genome is very large (Gaut & Doebley, 1997) and is especially known for its array of TEs; indeed it is from maize that McClintock (1950) first hypothesized that some genetic elements could be mobile.

Using bacterial artificial chromosome and fosmid clones, the genome of maize variety B73 was assembled, and revealed that long‐terminal repeat retrotransposons (LTRs) account for 74% of the genome. Proliferation of the LTRs was the primary reason why the maize genome is so expanded relative to other grasses (Schnable *et al*., 2009). Resequencing of 17 wild relatives, 23 traditional landraces and 35 improved maize lines and mapping back to the B73 reference genome suggested that introgression from wild relatives could be responsible for diversity recovery in maize following domestication and identified genes with diverse biological functions having been under selection during domestication (Hufford *et al*., 2012).

Although significant advances have been made in maize genome sequencing and population genomics, GWAS in maize is a challenge because LD decays within 2 kb. The development of the large NAM panel (McMullen *et al*., 2009) has significantly increased the efficiency of GWAS in maize, and loci associated with multiple agronomic traits have been identified (Table S2).

Using single‐molecule real‐time sequencing and high‐resolution optical mapping, an improved B73 genome was more recently assembled (Jiao *et al*., 2017). Due to significant structural variation among inbred lines, the genome of B73 alone is not sufficient to fully explain the variation among other inbred lines. Thus, the genomes of other inbred lines, including PH207 (Hirsch *et al*., 2016), W22 (Springer *et al*., 2018), Mo17 (Sun *et al*., 2018), HuangZaoSi (Li *et al*., 2019), small‐kernel (Yang *et al*., 2019) and B73‐Ab10, a variant of B73 containing Abnormal chromosome 10 (J. Liu *et al*., 2020), have been assembled. This and other ongoing work will facilitate increased understanding of maize genome diversity, as well as the breeding and improvement of maize.

## II. Genomics of underutilized crops to‐date

The genetic improvement of underutilized crops is, in part, constrained by limited genome resources. Recent developments in genome technology and the reduction of sequencing costs means genome‐scale research is no longer limited to major food crops (Table 1).

**Table 1:** Underutilized crops for which reference genomes are available; crops are organized into families (subfamilies) and the genome statistics are given.

Family	Species	Common name	Chromosome no.	Estimated genome size	Sequencing method^1^	Scaffold N50	Number of contigs	Assembled scaffolds size	References
Poaceae (Pooideae)	*Secale cereale*	Rye	2*n* = 2*x* = 14	7.92 Gb	H2000	9.4 kb	1581 707	2.80 Gb	Bauer *et al*. (2017)
	*Aegilops tauschii*	Wheat (D‐genome)	2*n* = 2*x* = 14	4.36 Gb	H2000	58.0 kb	179 145	4.23 Gb	Jia *et al*. (2013)
Poaceae (Chloridoideae)	*Eleusine coracana*	Finger millet	2*n* = 4*x* = 36	1.45 Gb	H4000, N500	23.7 kb	525 759	1.20 Gb	Hittalmani *et al*. (2017)
				1.5 Gb	NS500, PB	905.3 kb	2812 919	1.20 Gb	Hatakeyama *et al*. (2018)
	*Eragrostis tef*	Tef	2*n* = 4*x* = 40	772 Mb	H2000, 454	85.0 kb		672 Mb	Cannarozzi *et al*. (2014)
				622 Mb	PB, H4000, Hi‐C	1.6 Mb	1344	576 Mb	VanBuren *et al*. (2020)
Poaceae (Panicoideae)	*Setaria italica*	Foxtail millet	2*n* = 2*x* = 18	510 Mb	GAII	12.3 Mb		397 Mb	Bennetzen *et al*. (2012)
				490 Mb	GAII, H2000	1.0 Mb	16 903	423 Mb	Zhang *et al*. (2012)
	*Setaria viridis*	Green millet	2*n* = 2*x* = 18	500 Mb	PB, H2000	11.2 Mb	75	395 Mb	Mamidi *et al*. (2020)
				401 Mb	ONT, NS500	19.5 Mb	44	397 Mb	Thielen *et al*. (2020)
	*Cenchrus americanus*	Pearl millet	2*n* = 2*x* = 14	1.79 Gb	H2000, PB	885.0 kb	175 708	1.79 Gb	Varshney *et al*. (2017b)
	*Panicum miliaceum*	Broomcorn/Proso millet	2*n* = 4*x* = 36	887 Mb	PB, X‐ten, BN, Hi‐C	8.24 Mb	1308	848 Mb	Shi *et al*. (2019)
				1 Gb	X‐ten	89 kb	171 982	823 Mb	Ott *et al*. (2018)
				923 Mb	PB, H2500	46.7 Mb	5541	855 Mb	Zou *et al*. (2019)
	*Echinochloa crus‐galli*	Barnyard millet	2*n* = 6*x* = 54	1.38 Gb	H2000, PB	1.8Mb		1.27 Gb	Guo *et al*. (2017)
					PB, H4000	4.1 Mb		1.34 Gb	Ye *et al*. (2020)
	*Digitaria exilis*	Fonio millet	2*n* = 4*x* = 36	893Mb	H2500, No6000, Hi‐C, BN	10.7 Mb	29 155	716 Mb	Abrouk *et al*. (2020)
					PB, H3000, H4000	1.73 Mb	3329	761Mb	X. Wang *et al*. (2021)
	*Coix lacryma‐jobi*	Adlay/Job's tears	2*n* = 2*x* = 20	1.68 Gb	H2000				Cai *et al*. (2014)
					PB, H2500	2.24 Mb	4519	1.62 Gb	Guo *et al*. (2020)
					PB, H2500	594.3 kb	13 691	1.28 Gb	Kang *et al*. (2020)
					PB, H2000, BN, Hi‐C	14.0 Mb	3321	1.73 Gb	H. Liu *et al*. (2020)
	*Sorghum bicolor*	Sorghum	2*n* = 2*x* = 20	730 Mb	BAC	35 Mb	12 873	679 Mb	Paterson *et al*. (2009)
					BAC, H2500	68.7 Mb	2688	655 Mb	McCormick *et al*. (2018)
Fabaceae	*Cajanus cajan*	Pigeonpea	2*n* = 2*x* = 22	833 Mb	BAC, GAII, H2000	516.1 kb	173 708	606 Mb	Varshney *et al*. (2012)
					BAC, 454	14.0 kb	332 766	511 Mb	Singh *et al*. (2012)
	*Cicer arietinum*	Chickpea	2*n* = 2*x* = 16	738 Mb	BAC, GAII, 454	77.3 kb		520 Mb	Jain *et al*. (2013)
					BAC, H2000, BAC	40.0 Mb	62 619	532 Mb	Varshney *et al*. (2013)
					454, GAII	39.9 Mb	182 734	510 Mb	Parween *et al*. (2015)
	*Vigna radiata*	Mungbean/green gram	2*n* = 2*x* = 22	543 Mb	H2000, 454	1.52 Mb	180 372	431 Mb	Kang *et al*. (2014)
	*Vigna. angularis*	Adzuki bean	2*n* = 2*x* = 22	542 Mb	H2000	1.3 Mb	25 426	467 Mb	Yang *et al*. (2015)
				612 Mb	H2000, 454	703 kb	36 516	443 Mb	Kang *et al*. (2015)
	*Vigna unguiculata*	Cowpea	2*n* = 2*x* = 22	640 Mb	H2000, BAC, PB	16.4 Mb	765	519 Mb	Lonardi *et al*. (2019)
	*Lupins albus*	White lupin	2*n* = 2*x* = 50	584 Mb	PB, H2000, Hi‐C	18.7 Mb	3171	474 Mb	Xu *et al*. (2020)
					PB, H3000, BN	17.4 Mb		451 Mb	Hufnagel *et al*. (2020)
	*Lupinus angustifolius*	Narrow leafed lupin	2*n* = 2*x* = 40		H2500	703.2 kb	14 379	609 Mb	Hane *et al*. (2017)
					PB, H2000	30.8 Mb	123	616 Mb	P. Wang *et al*. (2021)
	*Vigna mungo*	Blackgram	2*n* = 2*x* = 22	574 Mb	X‐ten and ONT	1.4 Mb		475 Mb	Jegadeesan *et al*. (2021)
	*Trifolium subterraneum*	Subterranean clover	2*n* = 2*x* = 16	540 Mb	H2000, M, 454	287.6 kb	101 010	472 Mb	Hirakawa *et al*. (2016)
					BN	1.4 Mb	264	512 Mb	Kaur *et al*. (2017)
Polygonaceae	*Fagopyrum esculentum*	Common buckwheat	2*n* = 2*x* = 16	1.2 Gb	H2000	25.1 Mb		1.18 Gb	Yasui *et al*. (2016b)
	*Fagopyrum tataricum*	Tartary buckwheat	2*n* = 2*x* = 16	540 Mb	H2000, H2500, M, PB, GBS	550.7 kb	8778	489.3 Mb	Zhang *et al*. (2017)
Amaranthaceae	*Chenopodium quinoa*	Quinoa	2*n* = 4*x* = 36	1.5 Gb	H2500, PB	86.9 kb	110 092	1.1 Gb	Yasui *et al*. (2016a)
					PB, BN	3.8 Mb	4232	1.18 Gb	Jarvis *et al*. (2017)
					H2500, PB	1.2 Mb	10 795	1.3 Gb	Zou *et al*. (2017)
Euphorbiaceae	*Manihot esculentum*	Cassava	2*n* = 2*x* = 36	746 Mb	H2000, 454, BAC	67 kb		432 Mb	Wang *et al*. (2014)
	*Ricinus communis*	Castor bean	2*n* = 2*x* = 20	320 Mb	Plasmid, Sanger	561.4 kb	25 800	325 Mb	Chan *et al*. (2010)
Convolvulaceae	*Ipomoea batatas*	Sweet potato	2*n* = 6*x* = 90	4.4 Gb	H2500, N500, H4000, 454	200.7 kb	35 919	836 Mb	Yang *et al*. (2017)
Dioscoreaceae	*Dioscorea dumetorum*	Trifoliate yam	2*n* = 2*x* = 36	322 Mb	H1500, ONT	3.2 Mb	1172	485 Mb	Siadjeu *et al*. (2020)
	*Dioscorea rotundata*	White Guinea yam	2*n* = 2*x* = 40	579 Mb	H2500, BAC	2.1 Mb	4723	594 Mb	Tamiru *et al*. (2017)

^1^ Sequencing approaches are abbreviated as follows: 454, Roche 454; BAC, Bacterial Artificial Chromosome sequencing; BN, BioNano; GAII, Illumina Genome Analyzer II; H, Illumina Hiseq; Hi‐C, high‐throughput chromosome conformation capture; M, Illumina Miseq; N500, NextSeq 500; No6000, Novaseq6000; ONT, Oxford Nanopore Technologies; PB, PacBio; X‐ten, Illumina X‐ten.

### 1. Reference genome sequences for underutilized crops and cross‐crop comparisons

The Poaceae (grasses) is the second largest plant family, with *c*. 12 000 species. Besides the staple crops rice, maize, wheat and sugarcane, and some previously common cereals such as barley, oats and rye, Poaceae also contain many underutilized crops, including sorghum, foxtail millet, finger millet and broomcorn millet that all use C_4_ photosynthesis, in which photorespiratory losses induced by hot and arid environments are reduced. The conversion of C_3_ rice and wheat towards C_4_ photosynthesis is a long‐standing biotechnological goal. Comparative genomics has revealed that genes involved in C_4_ carbon fixation are all present in C_3_ plants (Zhang *et al*., 2012), and therefore the C_4_ pathway might have evolved from ancestral C_3_ isoforms. The panicoid grasses maize and sorghum show greater conservation of these genes compared to the pooid grasses rice and *Brachypodium* (Bennetzen *et al*., 2012). Furthermore, a tandem duplication of the carbonic anhydrase Caβ subfamily, which hydrates atmospheric CO_2_ to bicarbonate in the mesophyll, was found in C_4_ plants, potentially vital for C_4_ evolution (Zhang *et al*., 2012). Genome comparisons between underutilized C_4_ crops and the staple C_3_ crops in the Poaceae will provide new suggestions for the evolution of C_4_ photosynthesis, with the potential to improve the photorespiration efficiency and subsequent drought tolerance of other underutilized and staple crops.

The Fabaceae is the third‐largest plant family, including many agronomically important grain and forage species. Legumes can improve soil fertility through the fixing of atmospheric nitrogen via root nodule‐specific bacteria. The discovery of many genes involved in nitrogen fixing has been aided through the study of underutilized legume genomes (Jain *et al*., 2013; Lu *et al*., 2018; Zhuang *et al*., 2019). In addition, the legumes also contain species with unique nutritional features; for example, adzuki bean, widely cultivated in Asia, is referred to as the ‘weight loss bean’ due to its sweet taste but low caloric and fat content. Genomic comparisons with other legumes found that adzuki bean has fewer starch and fatty acid biosynthesis genes, which could play a role in its unique nutritional profile (Yang *et al*., 2015).

Several underutilized crops are advocated as worthy of investment because of their extreme stress resilience, often greater than staple crops (Massawe *et al*., 2015; Cullis & Kunert, 2017). Further comparative genomics in the Poaceae has identified numerous gene family expansions associated with stress tolerance in underutilized crops, and these might explain the high stress resistance in underutilized crops. Drought‐tolerant foxtail millet and sorghum (compared to drought‐susceptible rice and maize) contain expansions of stress response gene families, including those encoding cytochrome P450 proteins, expansins, lipid transfer proteins and several others, as well as miRNA169 targeting drought stress‐associated transcription factor nuclear factor‐YB (Paterson *et al*., 2009; The International Brachypodium Initiative, 2010). Tef, a drought‐tolerant cereal mainly distributed in Ethiopia, contains a tandem duplication of the nucleotidase/phosphatase *SAL1*, a gene family involved in drought tolerance, relative to other grasses investigated (Cannarozzi *et al*., 2014). Pearl millet possesses more members of cutin, suberin, wax biosynthetic and metabolite transporter genes, which might be responsible for the heat and drought tolerance in this underutilized crop (Varshney *et al*., 2017b). The number of BTB ubiquitin E3 ligases is greater in grasses than in *Arabidopsis*, and one subgroup, the BTB‐BACK subgroup, was only expanded in the underutilized cereal broomcorn millet (Zou *et al*., 2019), which may contribute to its excellent stress tolerance. Clearly, genome comparison of these underutilized crops will provide a new pool of stress‐targeted genes for well‐studied main crops. Similar results were found in the genus *Dioscorea*, in which draft genomes have been assembled for two yam species (Tamiru *et al*., 2017; Siadjeu *et al*., 2020), and phylogenetic analyses show that *Dioscorea* has more bulb‐type lectin genes than the Poaceae and *Arabidopsis*, with potential roles in the insecticidal properties of Guinea yam (Tamiru *et al*., 2017).

In summary, reference genomes of underutilized crops can help resolve the genetic basis of agronomic traits, especially as a (sometimes unique) resource for improving the photorespiratory efficiency, nutritional value and stress tolerance of related major food crops currently challenged by climate change.

### 2. Population genomics in underutilized crops

**Fig. 1:**
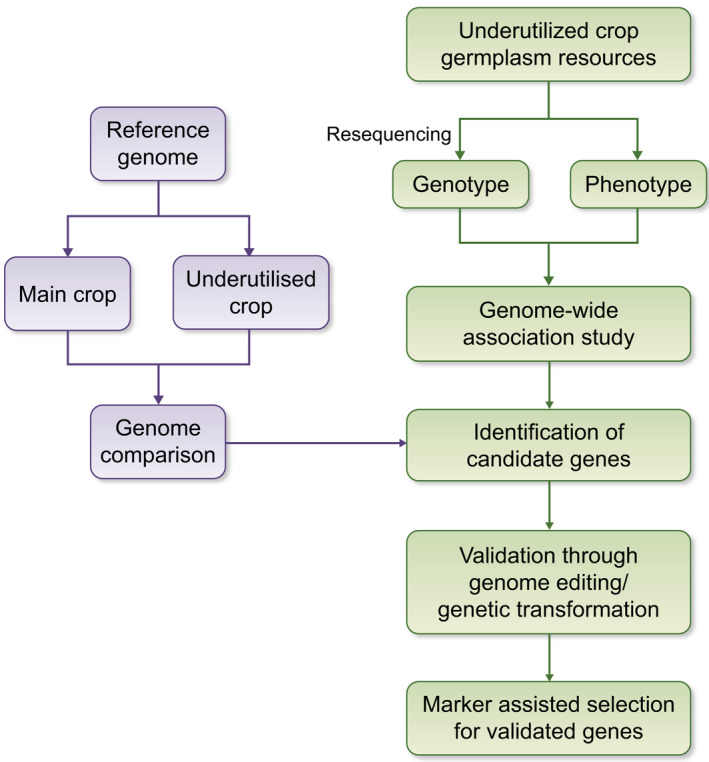
Schematic diagram highlighting the main sequencing and breeding approaches discussed in this article. First (left), by comparing the reference genomes of underutilized crops and staple/main crops, genome variants responsible for superior agronomic traits (such as specific resistance and nutrient quality or quantity) of underutilized crops could be explored. In addition (right), the combination of genome resequencing and phenotyping through genome‐wide association study could help to identify candidate genes responsible for agronomic traits of underutilized crops. Through genetic transformation or genome editing, the function of these candidate genes could be verified. Finally, by associating genomic data to phenotypic information of different accessions, germplasm resources can be effectively screened and bred by means of molecular marker‐assisted breeding and crossing, to improve the resistance and nutritional value of underutilized crops in addition to staple crops.

Population genomics of underutilized crops can help researchers to understand population structure and domestication history, as well as aid in identifying candidate genes modulating key agronomic traits through GWAS and to develop molecular markers for marker‐assisted breeding (MAB; Fig. 1).

#### Identifying crop wild relatives and untapped resources

The combination of genome resequencing and phylogenetic analysis enables us to better understand the population structure and identify wild relatives. For example, resequencing of 994 pearl millet lines identified four main clusters and pinpointed a West African origin for the crop (Varshney *et al*., 2017b). Resequencing of 166 fonio millet accessions and 17 proposed progenitors found a significant separation between cultivated and wild accessions, and the cultivated accessions were genetically closest to wild accessions from Southern Togo and West Guinea (Abrouk *et al*., 2020). Furthermore, diversity of the grain size gene *GS5* was significantly reduced in fonio millet cultivars (with larger grains than the wild progenitor), suggesting human selection on this locus during domestication. Resequencing of 510 accessions of Tartary buckwheat from the entire global distribution identified three major clades and indicated two geographically distinct domestication events (Zhang *et al*., 2021). Similar investigations in other underutilized crops have identified wild relatives and genetic subpopulations of the domesticates, for example adzuki bean (Yang *et al*., 2015), lupin (Hufnagel *et al*., 2020; P. Wang *et al*., 2021) and coix (H. Liu *et al*., 2020). Resequencing of Guinea yam and its potential wild relatives resolved a hybrid origin from a cross between the wild rainforest species *Dioscorea praehensilis* and the savanna species *D. abyssinica* (Sugihara *et al*., 2020).

#### GWAS and selection analyses

The high‐density SNP data obtained from population‐scale genome resequencing can be used to identify the genetic basis of adaptive traits through GWAS and selection analyses. From this, genetic markers can be designed and used for MAB. Beyond breeding, genetic modification‐type approaches can be used to insert the candidate genes from a stress‐tolerant or otherwise novel underutilized crop into a susceptible or trait‐lacking staple crop.

Seed size and weight are some of the most important traits of many crops, affecting price and milling qualities. GWAS of 368 cowpea accessions found candidate genes involved in endosperm development, embryo development and cell elongation associated with seed size, some of which also play a role in common bean (Lo *et al*., 2019). Similar analyses in castor bean identified candidate genes for seed traits that differentiate the wild and cultivated types (Xu *et al*., 2021). Recently, GWAS of Tartary buckwheat identified a mutation in the GCC *cis*‐element of an *AP2* transcription factor associated with grain weight (Zhang *et al*., 2021).

Underutilized crops can possess multiple agronomic traits that are not present in staple crops, and therefore present untapped resources for traits such as abiotic tolerances and novel nutrients. GWAS has been used to identify genes involved in tuber quality in cassava (S. Zhang *et al*., 2018) and anthocyanin content in mungbean (Noble *et al*., 2018). Regarding stress tolerance, genes involved in lateral root development, stress tolerance and phosphorus use efficiency of mungbean have been identified (Reddy *et al*., 2020).

Comparing wild and domesticated cassava genomes has identified selective sweeps in genes involved in photosynthesis, starch accumulation and stress response (Wang *et al*., 2014). Extending this to other crops will facilitate the development of markers associated with domestication‐related traits. Candidate genes identified in underutilized crops through GWAS can help improve the quality and stress tolerance not only of the underutilised crops but also of related staple crops, to better suit our needs in a changing climate.

Extended population genomics, for example using reduced representation technologies, can be used to link genetic markers to agronomic traits, without knowing the precise genetic basis of the trait. Markers linked to several diverse agronomic traits in foxtail millet have been identified using these approaches, for example coloration, leaf size and shape, grain yield and weight, and flowering time (Upadhyaya *et al*., 2015; Jaiswal *et al*., 2019). Similarly, QTL mapping approaches at sufficient density can provide marker–trait associations, for example markers associated with yield and flowering in pea (Annicchiarico *et al*., 2017) and dormancy in groundnut (Kumar *et al*., 2020).

Genomics‐assisted breeding of underutilized crops has been limited due to the lack of molecular markers linked to traits of interest, but the recent recognition of the importance of underutilized crops and the development of genome technology have clearly started to remedy this. The use of these markers to accelerate breeding (i.e. genomic selection) has been shown for a handful of underutilized crops (Ye & Fan, 2020).

### 3. Genetic transformation and gene editing in underutilized crops

In recent years, genetic engineering has been widely used to elucidate gene function and for crop improvement. Compared with traditional hybridization and crossing of varieties, genetic engineering could deliver agronomically useful traits into plants faster and in a more targeted manner. *Agrobacterium*‐based transformation systems are widely used for genetic transformation in plants, facilitating the integration of foreign gene copies into the host plant’s genome. Although *Agrobacterium* transformation has been successfully used for transformation in several major crops, the inherent limitations associated with resistance to *Agrobacterium* infection and their recalcitrance to *in vitro* regeneration limit the transformation of many orphan crops. At present, *Agrobacterium*‐mediated transformation has only been successful in shoot apex explants of finger millet (Ceasar & Ignacimuthu, 2011) and foxtail millet (Ceasar *et al*., 2017), callus derived from mature seeds of finger millet (Hema *et al*., 2014), green millet (Martins *et al*., 2015a; Nguyen *et al*., 2020), sorghum (Zhao *et al*., 2000; Belide *et al*., 2017) and foxtail millet (Santos *et al*., 2020), embryonic axis explants of pigeonpea (Ghosh *et al*., 2017), germinated seedlings of chickpea (Senthil *et al*., 2004), and hairy roots of chickpea (Aggarwal *et al*., 2018) and buckwheat (Mi *et al*., 2020). However, the recalcitrant tissue culture efficiency and occasional and unpredictable chimerism lower the efficiency of these tissue culture‐based methods.

Recently, using the floral‐dip *Agrobacterium*‐mediated transformation method, the wild ancestor of foxtail millet, green millet, was successfully transformed (Martins *et al*., 2015b). This is a significant advance because millets are model C_4_ grasses, and green millet it diploid, with a rapid life cycle, small genome size, simple growth requirements and high transformation efficiency.

Despite ongoing challenges of carrying out gene editing in even the best studied crops (Yang, 2020), CRISPR/Cas9‐based gene editing has been conducted in underutilized crops with relatively high tissue culture efficiency, including green millet (Weiss *et al*., 2020) and sorghum (Jiang *et al*., 2013; Che *et al*., 2018). These approaches will provide the necessary technical support for improving the efficiency in confirming the function of unique genes and the development of advantageous varieties of underutilized crops.

## III. The way forward

### 1. The successful transition from underutilized to mainstream

In the past 20 yr, a few previously underutilized crops, such as quinoa, chickpea and pigeonpea, have seen a significant boost in research and recognition. For these crops we have seen a parallel 20–500% increase in the area grown worldwide between the 1960s and 2010s (http://faostat.fao.org; accessed August 2021). Chickpea and pigeonpea were among the first underutilized crops to have their genomes sequenced (Varshney *et al*., 2012, 2013) with the (tetraploid) quinoa genome being made available more recently (Jarvis *et al*., 2017). Clearly the availability of genome sequence was a major stepping‐stone in resolving the genetic basis of adaptive and agronomic phenotypes in these crops.

Using pigeonpea as an exemplar, this crop was recognized as worthy of significant investment in the 1970s, with the Pigeonpea Genomics Initiative (PGI) established in 2006 (Varshney *et al*., 2010). After genome sequencing (Varshney *et al*., 2012), significant advances have been made in identifying genomic regions underlying adaptive traits that could be crossed between varieties using MAB (Varshney *et al*., 2017a), for example markers associated with sterility mosaic disease (Saxena *et al*., 2017a) and fusarium wilt (Saxena *et al*., 2017b). Genomic analysis has revealed fewer genes involved in lipid biosynthesis in pigeonpea than in soybean, and more cellulose synthesis genes, which together might underlie the biochemical and morphological differences between pigeonpea and other legumes (Singh *et al*., 2012). In addition, a pigeonpea gene involved in disease resistance was cloned and transferred to soybean, conferring resistance to Asian soybean rust (Kawashima *et al*., 2016), which would have been impossible without using the pigeonpea genome sequence.

More recent GWAS of nearly 300 pigeonpea accessions (Varshney *et al*., 2017a) identified dozens of associations and provided significant resources for MAB (Bohra *et al*., 2020). Pigeonpea is probably the only underutilized crop for which a pangenome has been sequenced (Zhao *et al*., 2020); this has 55 512 genes, compared to the reference genome (Varshney *et al*., 2012), which has only 53 612 (when annotated in exactly the same way as the pangenome). Using this pangenome, novel GWAS associations have been identified (Zhao *et al*., 2020), which were absent using the single reference genome (Varshney *et al*., 2017a). This further highlights the additional insights that can be made when a pangenome is made available.

Chickpea is grown and consumed worldwide, but 30 yr ago could have been considered underutilized. Although productivity has steadily increased, the development of accessions with greater yield, improved nutrition and stress resistance is essential to meet increasing demands. Comparative genomics of legumes has identified a lack of some resistance and nodulation genes, potential reasons for the low stress resistance (Jain *et al*., 2013; Varshney *et al*., 2013). Resequencing panels have identified genetic groups of cultivars (primarily the desi and kabuli types), identified the origin of the crop, and uncovered genes involved in drought tolerance and heat stress response through GWAS (Varshney *et al*., 2013, 2019).

Several other previously underutilized crops are seeing a revolution in their investigation, suggesting they are on the path to escaping some of the reasons they were previously underutilized. The following examples are case studies of crop species early on this trajectory and provide ideas to circumvent issues such as large genomes and examples of crops with unique attributes which have received investment.

For species with polyploid genomes, investigations of related diploids can shed light on agronomic traits. Oat is a nutritional crop containing abundant calcium, dietary fibre (especially β‐glucan) and unsaturated fatty acids (Joyce *et al*., 2019). Due to the cholesterol‐lowering properties and the antidiabetic effect of β‐glucan, oat has been widely used in adjuvant treatment of diabetes and cardiovascular disorders. The rotation of oat with other crops can improve soil structure and reduce diseases in other crops. This disease resistance has been attributed to the production of avenacins, specialized antifungal metabolites. Oat is allohexaploid, with a relatively large, highly repetitive and rearranged genome, and thus brings challenges for genome assembly. Current sequencing has mainly focused on wild diploid oats. For example, through genome assembly of the diploid extant progenitors, candidate genes regulating flowering time and disease resistance were identified (Maughan *et al*., 2019). Genome assembly of other diploid accessions identified a 12‐gene cluster responsible for avenacin biosynthesis, and this cluster was located in a subtelomeric region which may have formed since oat diverged from other crops (Li *et al*., 2021). These results shed light on the evolution of oat and will help in breeding oat varieties with modified and improved health benefits.

For other underutilized crops, they bring qualities and traits which are lacking in mainstream staple crops, and as such significant investment has begun to start their escape from being underutilized. Quinoa is one example, a crop of the Chenopodiaceae, which has been cultivated for *c*. 7000 yr. Its diverse environmental adaptability means it is grown from the sea level of Chile to altitudes above 4500 m in Bolivia (Suárez‐Estrella *et al*., 2018). Due to its extraordinary balance of essential amino acids, and abundant vitamins, minerals, dietary fibre and unsaturated fatty acids, it was recognized as a complete food and has attracted the attention of the scientific community (Filho *et al*., 2017). However, quinoa contains bitter and astringent antinutritional factors such as saponins. Although these substances are health‐promoting, their bitter taste has limited the utilization of quinoa (Suárez‐Estrella *et al*., 2018). Thus, selection of genotypes with low saponin content is one of the most important quinoa breeding objectives for the future. In addition, due to its outcrossing nature, genome assembly of quinoa was not trivial, requiring repeated self‐pollination to reduce heterozygosity (Yasui *et al*., 2016a). Analysis of the subsequently assembled quinoa genome (Yasui *et al*., 2016a) identified expansions of gene families involved in lysine, vitamins, polyphenol and betalain synthesis, as well as abscisic acid (ABA) signalling, which together may relate to the unique profile of nutritional and antinutritional factors and abiotic stress tolerance in quinoa (Yasui *et al*., 2016a; Zou *et al*., 2017).

Another example of an underutilized crop with novel attributes is buckwheat, a pseudocereal originating from and domesticated in China > 4000 yr ago (Zhang *et al*., 2021). This crop possesses an outstanding nutritional profile (especially flavonoids) and an excellent ability to grow under adverse climatic and soil conditions. The main cultivated species are common buckwheat and Tartary buckwheat. Similar to quinoa, being outcrossing makes genome sequencing of common buckwheat more challenging and requires repeated self‐pollination to reduce heterozygosity (Yasui *et al*., 2016b). In contrast to common buckwheat, the sequencing of Tartary buckwheat was relatively simple because of a smaller genome and because its is predominantly a selfer. Comparative genomics using the chromosome‐scale Tartary buckwheat genome revealed a whole‐genome duplication event after buckwheat divergence from sugar beet, with some evidence that this might play a role in buckwheat tolerance of extremely harsh environments (Zhang *et al*., 2017). Genome resequencing of Tartary buckwheat identified two independent domestication events, in southwestern and in northern China, which has resulted in the diversity of modern Tartary buckwheat varieties (Zhang *et al*., 2021). Candidate genes responsible for flavonoid biosynthesis were also identified and will help breeding of buckwheat with improved health and medical benefits.

Given these findings from the significant investment and the ongoing work in representative orphan crops (Roorkiwal *et al*., 2020), we feel encouraged that the resources and investment needed for these crops to be elevated to the national stage are in place. However, these are only the tip of the underutilized crop iceberg; dozens of underutilized crops have a single reference genome, and in some cases small resequencing panels (Table 1), but significant population and GWAS resources or pangenomes are absent for the vast majority.

### 2. What do we need and why? The advantages of going beyond a reference genome

To efficiently breed improved varieties of underutilized crops we need to have reliable linkage between genetic markers and traits of interest. Markers identified in a single QTL mapping experiment may not be reliable given that many QTL are only expressed in some environments (genotype × environment interaction) and do not always tightly define the genomic region (therefore the QTL spans dozens or hundreds of genes). More precision can be gleaned from LD mapping approaches (Thornsberry *et al*., 2001), including GWAS, which requires extensive panels of germplasm and high marker density. A reference genome is an asset to begin to understand important and adaptive phenotypes in underutilized crops, yet it is becoming clear that significant advances in breeding improved varieties are only possible when the genomic variants are identified, thus requiring a population of genomes and potentially a pangenome.

#### Quality trait‐marker linkage

**Fig. 2:**
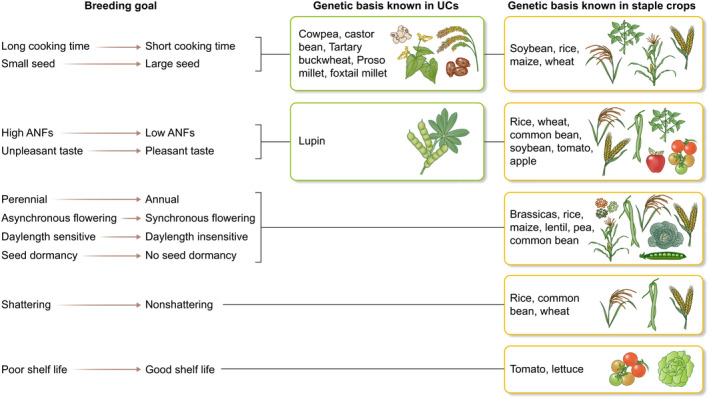
Schematic diagram highlighting common breeding targets for any crop, and whether the genetic basis has been investigated in underutilized crops (UCs) and/or staple crops. ANF, antinutrient factor.

One main advantage to having population‐level sequencing is to tie this to trait data using GWAS‐type approaches. This is an efficient way to start to narrow down the genetic basis of quantitative traits such as yield, seed and organ size, plant stature, etc., all traits which need to be optimized to ensure a crop is cost‐effective to be grown at scale (Fig. 2). A reference genome is an asset, but without the resequencing (or high‐density SNP genotyping), GWAS cannot be done. Examples of GWAS‐style analyses in the underutilized crops cowpea, castor bean, Tartary buckwheat, cassava and mungbean are given above.

Using reduced‐representation technology (e.g. genotyping‐by‐sequencing, or restriction site‐associated DNA sequencing) is an efficient way to screen large numbers of genetic loci throughout the genome at lower cost than resequencing. The downside is that unless LD extends for very long distances, most markers will be so widely spaced that they will be too far from the underlying causative loci to be associated. Nevertheless, these approaches have yielded marker‐trait associations (MTAs) in some underutilized crops and pave the way for more in‐depth analyses. For example, in Proso millet (*Panicum milaceum* L.) 13 MTAs for seven traits were resolved, but no MTAs were found for another nine traits (Boukail *et al*., 2021). In foxtail millet, 81 MTAs for 10 traits were resolved, but most did not pass false discovery rate correction (Jaiswal *et al*., 2019), and in Kersting’s groundnut, 10 MTAs for five traits were resolved (Akohoue *et al*., 2020). It is important to bear in mind that because of the marker spacing, partly due to the location of cut sites in the genome, and partly because of uneven sequencing coverage across loci (Beissinger *et al*., 2013), these studies are likely to report only a subset of genomic loci involved in the traits of interest.

#### Cross‐crop analyses

Whilst underutilized crops may lack some desirable traits (fast growth, ease of harvest, high harvest index), or have additional phenotypes that are unwanted (antinutrients, perenniality), significant advances have been made in understanding the genetic basis of many of these traits in other crops (Fig. 2). Thus, where these traits have been studied in detail, there may be candidate genes that can be followed up if population resequencing or pangenomic data are available in the underutilized crop.

Several underutilized crops would benefit from having more predictable flowering/fruiting or being adapted to novel environments where the daylength is different. The legume lablab is typically a short‐day plant, and therefore expansion outside its native tropical latitudes is unlikely to be successful (Sennhenn *et al*., 2017). Bambara groundnut, another tropical underutilized legume, is typically short‐day although a few semiimproved varieties can be grown further from the equator, but it is acknowledged that this is still a barrier to more widespread adoption (Mayes *et al*., 2019). Daylength response (and therefore flowering time) is relatively well studied in staple crops, including rice, maize and the typically long‐day legumes, lentil and pea (Hung *et al*., 2012; Weller *et al*., 2012; Itoh & Izawa, 2013), offering candidate genes for the development of underutilized cereal and legume varieties for adaptation to nonnative latitudes. Candidate genes, or genomic regions, underlying annual vs perennial growth have been identified in Brassicaceae species (Heidel *et al*., 2016; Kiefer *et al*., 2017); this is another trait which might help the adoption of underutilized crops.

An often‐cited reason for the poor adoption, or decline in use, of underutilized crops is their antinutrient content. Antinutrient factors (ANFs) inhibit the uptake of beneficial minerals and vitamins, so a high‐nutrient crop with high ANF content will have low nutrient bioavailability. This is especially the case in legumes where several ANFs have been identified that affect iron, zinc and protein uptake. Whilst cooking and fermentation can reduce the presence of these compounds (e.g. Samtiya *et al*., 2020), these take time or energy (e.g. fuel for cooking). However, ANFs are usually vital for crop disease resistance, and therefore breeding for high ANF during the growth period coupled with low ANF in the maturation period would clearly be advantageous. Progress has been made in understanding the genetic basis of these traits (Campion *et al*., 2013; Sparvoli & Cominelli, 2015), opening the door for understanding the genetic basis of these traits in underutilized crops.

There are other traits which make underutilized crops less attractive as a choice for a farmer or the consumer, for example poor shelf‐life, unpleasant taste or lengthy cooking times (and an increase cost for fuel). Genes involved in shelf‐life in tomato have been elucidated (Casals *et al*., 2012; L. Zhang *et al*., 2018), along with QTL for alkaloid content in lupin (Rychel & Książkiewicz, 2019) and for seed hardness, and therefore cooking time, in legumes (Sandhu *et al*., 2018; Diaz *et al*., 2021).

Relatedly, many underutilized crops are known for their extreme resilience phenotypes. Any analysis of the genetic basis of drought or heat tolerance in any underutilized crops, probably requiring population sequencing for GWAS, for example, will be of significant value to other more mainstream crops. This could identify novel alleles or even undercover novel genes and pathways involved in these climate‐change‐relevant tolerances. The sequencing of one reference genome of an underutilized crop cannot offer this.

Population‐level resequencing mapped to one reference will not be able to examine the fraction of the genome that is only present in some accessions (presence–absence variation lacking from the sequenced reference, which would only be identified in a pangenome). This problem could be underestimated for underutilized crops where variation in genome size might not be recognized; for example, the underutilized legume lablab was probably domesticated twice (Robotham & Chapman, 2015; Maass *et al*., 2017), and the two gene pools differ in genome size by *c*. 20% (MAC, unpublished).

#### Next steps

We propose that efforts should be made not only to generate a reference genome but also to carry out population‐level sequencing and pangenomics. In parallel we encourage the continued collection and long‐term archiving of seed resources, and addressing the challenges associated with archiving the required indigenous knowledge associated with these under‐investigated species (Mabhaudhi *et al*., 2017; Kamenya *et al*., 2021). Researchers should make data free to use, and collaborations between institutes worldwide should be encouraged to expedite the production of results and limiting unnecessary overlap and wasted resources.

Whilst the cost and time implications of multiple reference genomes, resequencing and collecting global germplasm are not trivial, we believe that, given the climate crisis and the need to fast‐track the development of mainstream and novel crops, this is the most reliable way to ensure that underutilized crops are investigated to the depth at which reliable and meaningful data can be used. It is likely that some underutilized crops hold vital genetic variants to help the human population combat food insecurity in the next few decades; this genetic erosion is under‐investigated even in staple crops (Khoury *et al*., 2022). Without fully investigating underutilized crop genomes, we do not know where these variants lie, and if we delay too long, we may lose alleles, varieties and crops entirely.

## Author contributions

MAC, YH and MZ researched the topic and wrote the manuscript.

## Supporting information


**Table S1** List of important traits dissected by GWAS in rice.
**Table S2** List of important traits dissected by GWAS in maize.
Please note: Wiley Blackwell are not responsible for the content or functionality of any Supporting Information supplied by the authors. Any queries (other than missing material) should be directed to the *New Phytologist* Central Office.
