# Supplementary material for: Beyond a reference genome: pangenomes and population genomics of underutilized and orphan crops for future food and nutrition security
Source: New Phytol. 2022 Mar 22;234(5):1583–97. doi: 10.1111/nph.18021 (PMC9994440; doi:10.1111/nph.18021)
Supplement: Supplementary file 1 — Table S1 List of important traits dissected by GWAS in rice. Table S2 List of important traits dissected by GWAS in maize. Please note: Wiley Blackwell are not responsible for the content or functionality of any Supporting Information supplied by the authors. Any queries (other than missing material) should be directed to the New Phytologist Central Office. [file NPH-234-1583-s001.pdf]

***New Phytologist* Supporting Information**

Article title: **Beyond a reference genome: pangenomes and population genomics of underutilised and orphan crops for future food and nutrition security**

Authors: **Mark A. Chapman, Yuqi He and Meiliang Zhou**

Article acceptance date: 22 January 2022

The following Supporting Information is available for this article:

Table S1. List of important traits dissected by GWAS in rice

Table S2. List of important traits dissected by GWAS in maize

Table S1. List of important traits dissected by GWAS in rice

Reference	Population	Loci associated with traits
Huang et al. 2010	373 indica accessions	3 loci associated with tiller number, 2 loci associated with grain width, 5 loci associated with grain length, 2 loci associated with spikelet number, 1 locus associated with gelatinization temperature, 3 loci associated with amylose content, 2 loci associated with apiculus color, 3 loci associated with pericarp color, 2 loci associated with 2 hull color, 7 loci associated with heading date, 4 loci associated with drought tolerance, 3 loci associated with seed shattering degree
Huang et al. 2012	950 rice accessions	2 loci associated with gelatinization temperature, 1 locus associated with hull color, 5 loci associated with grain length, 1 locus associated with grain number, 4 loci associated with grain width, 16 loci associated with heading date, 4 loci associated with amylose content, 4 associated with pericarp color, 3 associated with protein content, 4 associated with grain weight, 1 associated with apiculus color
Chen et al. 2014	529 rice accessions	634 loci associated with 598 leave metabolites
Yang et al. 2014b	529 accession	5 loci associated with shoot fresh weight, 3 loci associated with shoot dry weight, 22 loci associated with plant height, 1 locus associated with tillers number, 7 loci associated with green leaves area, 11 loci associated with plant compactness, 11 loci associated with grain weight, 1 locus associated with filled spikelets number, 1 locus associated with grain fertility, 21 loci associated with grain length, 10 loci associated with grain length/width ratio, 28 loci associated with grain projected area, 19 loci associated with grain width, 1 locus associated with yield per plant
Wang et al. 2015	529 accession	46 loci associated with chlorophyll content
Chen et al. 2016	502 rice accessions	476 loci associated with 331 grain metabolites
Crowell et al. 2016	242 accessions	489 loci associated with 49 panicle traits
Meyer et al. 2016	93 African rice landraces	11 loci associated with salt tolerance
Si et al. 2016	381 japonica varieties	1 locus associated with grain length
Wang et al. 2016a	203 accessions	1 locus associated with grain length, 1 locus associated with amylose content in rice kernels, 2 loci associated with pericarp color
Yano et al. 2016	176 japonica varieties	12 loci associated with spikelet number, 1 locus associated with panicle number per plant, 5 loci associated with heading date, 3 loci associated with panicle length, 47 loci associated with plant height, 9 loci associated with leaf blade width, 50 loci associated with awn length
Duan et al. 2017	102 indica varieties	3 loci associated with grain width
Gong et al. 2017	10084 F2 lines	12 loci associated with grain length-to-width ratio, 11 loci associated with chalky grain rate
Zhou et al. 2017	533 accessions	23 loci associated with stigma exsertion and related floral traits
Dong et al. 2018	529 rice accessions	62 loci associated with flag leaf angle
Guo et al. 2018	507 rice accessions	470 loci associated with drought resistance
Sun et al. 2018	510 accessions	3 loci associated with mesocotyl length
Xiao et al. 2018	1,033 accessions	5 loci associated with cold tolerance at the booting stage, 8 loci associated with cold tolerance at the seedling stage

Liu et al. 2019	208 rice accessions	16 loci associated with mesocotyl length
Ma et al. 2019	270 rice accessions	5 loci associated with grain length, 4 loci associated with grain width
Tang et al. 2019	117 rice accessions	2 loci associated with nitrogen use efficiency
Yan et al. 2019	127 rice cultivars	12 loci associated with grain Cd accumulation
Zhang et al. 2019	510 accessions	33 loci associated with callus induction rate, 31 loci associated with callus induction speed, 24 loci associated with time of the first callus appearance
Liu et al. 2020	584 rice accessions	27 loci associated with rice blast resistance
Neang et al. 2020	296 accessions	1 locus associated with salt removal in leaf sheaths
Yuan et al. 2020	664 rice accessions	21 loci associated with salt tolerance
Zhang et al. 2020	137 rice accessions	2 loci associated with maximum root length, 2 loci associated with total root weight
Liu et al. 2021	110 rice accessions	1 locus associated with tillering response to nitrogen

Table S2. List of important traits dissected by GWAS in maize

Reference	Population	Loci associated with traits
Buckler et al. 2009	5000 NAM population	333 loci associated with flowering time
Tian et al. 2011	4892 NAM population	36 loci associated with leaf length, 34 loci associated with leaf width, 30 loci associated with upper leaf angle
Poland et al. 2011	5000 NAM population	29 loci associated with northern leaf blight resistance
Kump et al. 2011	5000 NAM population	32 loci associated with southern leaf blight resistance
Jiao et al. 2012	278 inbred lines	5 loci associated with silk color, 5 loci associated with days to anthesis, 5 loci associated with cob color
Cook et al. 2012	4699 NAM population and 282 inbred lines	21 loci associated with starch content, 26 loci associated with protein content, 22 loci associated with oil content
Li et al. 2013	368 inbred lines	74 loci associated with oil concentration or composition
Strigens et al. 2013	375 inbred lines	19 loci associated with chilling tolerance
Yang et al. 2013	368 inbred lines	48 loci associated with flowering time
Wen et al. 2014	368 inbred lines	1256 loci associated with 501 metabolites in maize kernel
Olukolu et al. 2014	3381 NAM population	44 loci associated with hypersensitive defense response
Thirunavukkarasu et al. 2014	240 inbred lines	28 loci associated with stomatal closure, 15 loci associated with flowering, 5 loci associated with root development, 4 loci associated with detoxification, 2 loci associated with reduced water potential
Yang et al. 2014a	513 inbred lines	6 loci associated with ear length, 2 loci associated with tassel main axis length, 1 locus associated with plant height, 1 locus associated with kernel width, 1 locus associated with ear leaf width
Benke et al. 2015	267 inbred lines	35 loci associated with iron homeostasis
Dell'Acqua et al. 2015	529 inbred lines	3 loci associated with grain yield and 3 loci associated with flowering time
Leiboff et al. 2015	384 inbred lines	51 loci associated with shoot apical meristem volume
Mao et al. 2015	368 inbred lines	1 locus associated with drought tolerance
Mammadov et al. 2015	300 inbred lines	4 loci associated with gray leaf spot resistance
Cui et al. 2016	508 inbred lines	5 loci associated with hush number, 1 locus associated with hush width, 3 loci associated with hush thickness
Li et al. 2016	258 inbred lines	21 loci associated with plant height; 20 loci associated with ear height
Wang et al. 2016b	368 inbred lines	37 loci associated with drought tolerance
Liu et al. 2016	263 inbred lines	4 loci associated with starch content in maize kernels
Adewale et al. 2020	132 inbred lines	11 loci associated with Striga resistance

Literature Cited in Tables S1 and S2

Adewale SA, Badu-Apraku B, Akinwale RO, Paterne AA, Gedil M, Garcia-Oliveira AL. 2020. Genome-wide association study of Striga resistance in early maturing white tropical maize inbred lines. *BMC Plant Biology* **20**(1): 203.

Benke A, Urbany C, Stich B. 2015. Genome-wide association mapping of iron homeostasis in the maize association population. *BMC Genetics* **16**: 1.

Buckler ES, Holland JB, Bradbury PJ, Acharya CB, Brown PJ, Browne C, Ersoz E, Flint-Garcia S, Garcia A, Glaubitz JC, et al. 2009. The Genetic Architecture of Maize Flowering Time. *Science* **325**(5941): 714-718.

Chen W, Gao Y, Xie W, Gong L, Lu K, Wang W, Li Y, Liu X, Zhang H, Dong H, et al. 2014. Genome-wide association analyses provide genetic and biochemical insights into natural variation in rice metabolism. *Nature Genetics* **46**(7): 714-721.

Chen W, Wang W, Peng M, Gong L, Gao Y, Wan J, Wang S, Shi L, Zhou B, Li Z, et al. 2016. Comparative and parallel genome-wide association studies for metabolic and agronomic traits in cereals. *Nat Commun* **7**: 12767.

Cook JP, McMullen MD, Holland JB, Tian F, Bradbury P, Ross-Ibarra J, Buckler ES, Flint-Garcia SA. 2012. Genetic architecture of maize kernel composition in the nested association mapping and inbred association panels. *Plant Physiology* **158**(2): 824-834.

Crowell S, Korniliev P, Falcão A, Ismail A, Gregorio G, Mezey J, McCouch S. 2016. Genome-wide association and high-resolution phenotyping link *Oryza sativa* panicle traits to numerous trait-specific QTL clusters. *Nat Commun* **7**: 10527.

Cui Z, Luo J, Qi C, Ruan Y, Li J, Zhang A, Yang X, He Y. 2016. Genome-wide association study (GWAS) reveals the genetic architecture of four husk traits in maize. *BMC Genomics* **17**(1): 946.

Dell'Acqua M, Gatti DM, Pea G, Cattonaro F, Coppens F, Magris G, Hlaing AL, Aung HH, Nelissen H, Baute J, et al. 2015. Genetic properties of the MAGIC maize population: a new platform for high definition QTL mapping in Zea mays. *Genome Biology* **16**(1): 167.

Dong H, Zhao H, Li S, Han Z, Hu G, Liu C, Yang G, Wang G, Xie W, Xing Y. 2018. Genome-wide association studies reveal that members of bHLH subfamily 16 share a conserved function in regulating flag leaf angle in rice (*Oryza sativa*). *PLoS Genetics* **14**(4): e1007323.

Duan P, Xu J, Zeng D, Zhang B, Geng M, Zhang G, Huang K, Huang L, Xu R, Ge S, et al. 2017. Natural Variation in the Promoter of GSE5 Contributes to Grain Size Diversity in Rice. *Molecular Plant* **10**(5): 685-694.

Gong J, Miao J, Zhao Y, Zhao Q, Feng Q, Zhan Q, Cheng B, Xia J, Huang X, Yang S, et al. 2017. Dissecting the Genetic Basis of Grain Shape and Chalkiness Traits in Hybrid Rice Using Multiple Collaborative Populations. *Molecular Plant* **10**(10): 1353-1356.

Guo Z, Yang W, Chang Y, Ma X, Tu H, Xiong F, Jiang N, Feng H, Huang C, Yang P, et al. 2018. Genome-Wide Association Studies of Image Traits Reveal Genetic Architecture of Drought Resistance in Rice. *Molecular Plant* **11**(6): 789-805.

Huang X, Kurata N, Wei X, Wang ZX, Wang A, Zhao Q, Zhao Y, Liu K, Lu H, Li W, et al. 2012. A map of rice genome variation reveals the origin of cultivated rice. *Nature* **490**(7421): 497-501.

Huang X, Wei X, Sang T, Zhao Q, Feng Q, Zhao Y, Li C, Zhu C, Lu T, Zhang Z, et al. 2010. Genome-wide association studies of 14 agronomic traits in rice landraces. *Nature Genetics* **42**(11): 961-967.

Jiao Y, Zhao H, Ren L, Song W, Zeng B, Guo J, Wang B, Liu Z, Chen J, Li W, et al. 2012. Genome-wide genetic changes during modern breeding of maize. *Nature Genetics* **44**(7): 812-815.

Kump KL, Bradbury PJ, Wisser RJ, Buckler ES, Belcher AR, Oropeza-Rosas MA, Zwonitzer JC, Kresovich S, McMullen MD, Ware D, et al. 2011. Genome-wide association study of quantitative resistance to southern leaf blight in the maize nested association mapping population. *Nature Genetics* **43**(2): 163-168.

Leiboff S, Li X, Hu HC, Todt N, Yang J, Li X, Yu X, Muehlbauer GJ, Timmermans MC, Yu J, et al. 2015. Genetic control of morphometric diversity in the maize shoot apical meristem. *Nat Commun* **6**: 8974.

Li H, Peng Z, Yang X, Wang W, Fu J, Wang J, Han Y, Chai Y, Guo T, Yang N, et al. 2013. Genome-wide association study

dissects the genetic architecture of oil biosynthesis in maize kernels. *Nature Genetics* **45**(1): 43-50.

Li X, Zhou Z, Ding J, Wu Y, Zhou B, Wang R, Ma J, Wang S, Zhang X, Xia Z, et al. 2016. Combined Linkage and Association Mapping Reveals QTL and Candidate Genes for Plant and Ear Height in Maize. *Frontiers in Plant Science* **7**: 833.

Liu H, Zhan J, Li J, Lu X, Liu J, Wang Y, Zhao Q, Ye G. 2019. Genome-wide Association Study (GWAS) for Mesocotyl Elongation in Rice (*Oryza sativa* L.) under Multiple Culture Conditions. *Genes (Basel)* **11**(1): 49.

Liu MH, Kang H, Xu Y, Peng Y, Wang D, Gao L, Wang X, Ning Y, Wu J, Liu W, et al. 2020. Genome-wide association study identifies an NLR gene that confers partial resistance to *Magnaporthe oryzae* in rice. *Plant Biotechnology Journal* **18**(6): 1376-1383.

Liu N, Xue Y, Guo Z, Li W, Tang J. 2016. Genome-Wide Association Study Identifies Candidate Genes for Starch Content Regulation in Maize Kernels. *Frontiers in Plant Science* **7**: 1046.

Liu Y, Wang H, Jiang Z, Wang W, Xu R, Wang Q, Zhang Z, Li A, Liang Y, Ou S, et al. 2021. Genomic basis of geographical adaptation to soil nitrogen in rice. *Nature* **590**(7847): 600-605.

Ma X, Feng F, Zhang Y, Elesawi IE, Xu K, Li T, Mei H, Liu H, Gao N, Chen C, et al. 2019. A novel rice grain size gene OsSNB was identified by genome-wide association study in natural population. *PLoS Genetics* **15**(5): e1008191.

Mammadov J, Sun X, Gao Y, Ochsenfeld C, Bakker E, Ren R, Flora J, Wang X, Kumpatla S, Meyer D, et al. 2015. Combining powers of linkage and association mapping for precise dissection of QTL controlling resistance to gray leaf spot disease in maize (*Zea mays* L.). *BMC Genomics* **16**: 916.

Mao H, Wang H, Liu S, Li Z, Yang X, Yan J, Li J, Tran LS, Qin F. 2015. A transposable element in a NAC gene is associated with drought tolerance in maize seedlings. *Nat Commun* **6**: 8326.

Meyer RS, Choi JY, Sanches M, Plessis A, Flowers JM, Amas J, Dorph K, Barretto A, Gross B, Fuller DQ, et al. 2016. Domestication history and geographical adaptation inferred from a SNP map of African rice. *Nature Genetics* **48**(9): 1083-1088.

Neang S, de Ocampo M, Egdane JA, Platten JD, Ismail AM, Seki M, Suzuki Y, Skoulding NS, Kano-Nakata M, Yamauchi A, et al. 2020. A GWAS approach to find SNPs associated with salt removal in rice leaf sheath. *Annals of Botany* **126**(7): 1193-1202.

Olukolu BA, Wang GF, Vontimitta V, Venkata BP, Marla S, Ji J, Gachomo E, Chu K, Negeri A, Benson J, et al. 2014. A genome-wide association study of the maize hypersensitive defense response identifies genes that cluster in related pathways. *PLoS Genetics* **10**(8): e1004562.

Poland JA, Bradbury PJ, Buckler ES, Nelson RJ. 2011. Genome-wide nested association mapping of quantitative resistance to northern leaf blight in maize. *Proc Natl Acad Sci U S A* **108**(17): 6893-6898.

Si L, Chen J, Huang X, Gong H, Luo J, Hou Q, Zhou T, Lu T, Zhu J, Shangguan Y, et al. 2016. *OsSPL13* controls grain size in cultivated rice. *Nature Genetics* **48**(4): 447-456.

Strigens A, Freitag NM, Gilbert X, Grieder C, Riedelsheimer C, Schrag TA, Messmer R, Melchinger AE. 2013. Association mapping for chilling tolerance in elite flint and dent maize inbred lines evaluated in growth chamber and field experiments. *Plant Cell Environ* **36**(10): 1871-1887.

Sun S, Wang T, Wang L, Li X, Jia Y, Liu C, Huang X, Xie W, Wang X. 2018. Natural selection of a GSK3 determines rice mesocotyl domestication by coordinating strigolactone and brassinosteroid signaling. *Nat Commun* **9**(1): 2523.

Tang W, Ye J, Yao X, Zhao P, Xuan W, Tian Y, Zhang Y, Xu S, An H, Chen G, et al. 2019. Genome-wide associated study identifies NAC42-activated nitrate transporter conferring high nitrogen use efficiency in rice. *Nat Commun* **10**(1): 5279.

Thirunavukkarasu N, Hossain F, Arora K, Sharma R, Shiriga K, Mittal S, Mohan S, Namratha PM, Dogga S, Rani TS, et al. 2014. Functional mechanisms of drought tolerance in subtropical maize (*Zea mays* L.) identified using genome-wide association mapping. *BMC Genomics* **15**(1): 1182.

Tian F, Bradbury PJ, Brown PJ, Hung H, Sun Q, Flint-Garcia S, Rocheford TR, McMullen MD, Holland JB, Buckler ES. 2011. Genome-wide association study of leaf architecture in the maize nested association mapping population. *Nature Genetics* **43**(2): 159-162.

Wang H, Xu X, Vieira FG, Xiao Y, Li Z, Wang J, Nielsen R, Chu C. 2016. The Power of Inbreeding: NGS-Based GWAS of Rice Reveals Convergent Evolution during Rice Domestication. *Molecular Plant* **9**(7): 975-985.

Wang Q, Xie W, Xing H, Yan J, Meng X, Li X, Fu X, Xu J, Lian X, Yu S, et al. 2015. Genetic Architecture of Natural Variation in Rice Chlorophyll Content Revealed by a Genome-Wide Association Study. *Molecular Plant* **8**(6): 946-957.

Wang X, Wang H, Liu S, Ferjani A, Li J, Yan J, Yang X, Qin F. 2016. Genetic variation in *ZmVPP1* contributes to drought tolerance in maize seedlings. *Nature Genetics* **48**(10): 1233-1241.

Wen W, Li D, Li X, Gao Y, Li W, Li H, Liu J, Liu H, Chen W, Luo J, et al. 2014. Metabolome-based genome-wide association study of maize kernel leads to novel biochemical insights. *Nat Commun* **5**: 3438.

Xiao N, Gao Y, Qian H, Gao Q, Wu Y, Zhang D, Zhang X, Yu L, Li Y, Pan C, et al. 2018. Identification of Genes Related to Cold Tolerance and a Functional Allele That Confers Cold Tolerance. *Plant Physiology* **177**(3): 1108-1123.

Yan H, Xu W, Xie J, Gao Y, Wu L, Sun L, Feng L, Chen X, Zhang T, Dai C, et al. 2019. Variation of a major facilitator superfamily gene contributes to differential cadmium accumulation between rice subspecies. *Nat Commun* **10**(1): 2562.

Yang N, Lu Y, Yang X, Huang J, Zhou Y, Ali F, Wen W, Liu J, Li J, Yan J. 2014. Genome wide association studies using a new nonparametric model reveal the genetic architecture of 17 agronomic traits in an enlarged maize association panel. *PLoS Genetics* **10**(9): e1004573.

Yang Q, Li Z, Li W, Ku L, Wang C, Ye J, Li K, Yang N, Li Y, Zhong T, et al. 2013. CACTA-like transposable element in ZmCCT attenuated photoperiod sensitivity and accelerated the postdomestication spread of maize. *Proceedings of the National Academy of Sciences of the United States of America* **110**(42): 16969-16974.

Yang W, Guo Z, Huang C, Duan L, Chen G, Jiang N, Fang W, Feng H, Xie W, Lian X, et al. 2014. Combining high-throughput phenotyping and genome-wide association studies to reveal natural genetic variation in rice. *Nat Commun* **5**: 5087.

Yano K, Yamamoto E, Aya K, Takeuchi H, Lo PC, Hu L, Yamasaki M, Yoshida S, Kitano H, Hirano K, et al. 2016. Genome-wide association study using whole-genome sequencing rapidly identifies new genes influencing agronomic traits in rice. *Nature Genetics* **48**(8): 927-934.

Yuan J, Wang X, Zhao Y, Khan NU, Zhao Z, Zhang Y, Wen X, Tang F, Wang F, Li Z. 2020. Genetic basis and identification of candidate genes for salt tolerance in rice by GWAS. *Sci Rep* **10**(1): 9958.

Zhang H, San ML, Jang SG, Lee JH, Kim NE, Lee AR, Park SY, Cao FY, Chin JH, Kwon SW. 2020. Genome-Wide Association Study of Root System Development at Seedling Stage in Rice. *Genes (Basel)* **11**(12): 1395.

Zhang Z, Zhao H, Li W, Wu J, Zhou Z, Zhou F, Chen H, Lin Y. 2019. Genome-wide association study of callus induction variation to explore the callus formation mechanism of rice. *J Integr Plant Biol* **61**(11): 1134-1150.

Zhou H, Li P, Xie W, Hussain S, Li Y, Xia D, Zhao H, Sun S, Chen J, Ye H, et al. 2017. Genome-wide Association Analyses Reveal the Genetic Basis of Stigma Exsertion in Rice. *Molecular Plant* **10**(4): 634-644.
